# Understanding perception and acceptance of Sinopharm vaccine and vaccination against COVID–19 in the UAE

**DOI:** 10.1186/s12889-021-11620-z

**Published:** 2021-08-30

**Authors:** Faheem Ahamed, Subhashini Ganesan, Anila James, Walid Abbas Zaher

**Affiliations:** 1 G42 Health Care, Masdar City, Abu Dhabi UAE; 2 YouGov, Canyan Business center, Barsha Heights, Dubai, UAE; 3 College of Medicine and Health Sciences United Arab Emirates University (UAEU), Al Ain, UAE

**Keywords:** Vaccine hesitancy, COVID-19, SARS-CoV-2 vaccine, Vaccine safety

## Abstract

**Background:**

In the current COVID-19 pandemic, the world has reached an important milestone where vaccinations are discovered and are proven to be effective against SARS-COV-2 infections. Though vaccines against COVID-19 are now available, around the globe there is some hesitancy in getting the vaccine. This hesitancy to get vaccinated against COVID-19 is a complex phenomenon with various factors playing a role. This study aims at understanding the perception and expectations of the people about COVID-19 vaccine and the factors influencing the vaccine acceptance. This information is crucial to challenge vaccine hesitancy and to win the combat against the COVID-19 Pandemic through voluntary vaccine efforts.

**Methods:**

A cross-sectional survey among the residents of the UAE to understand the expectations and perception on vaccination against COVID-19. The survey was conducted online, and the survey design included participant samples to be representative of UAE’s demographics. The results of the survey were analysed with various demographical variables of interest.

**Results:**

The survey showed that people were more likely to get vaccinated when vaccines are (i) endorsed by trusted government health authorities, (ii) recommended by physicians and family doctors, and (iii) the merits are effectively communicated through government websites and trusted news channels. Availability of vaccines at multiple sites and providing vaccines free of charges are likely to improve the rate of vaccination. The perceptions, expectations and the motivational factors needed for people to get vaccinated differed with age, gender, marital status, income level, and employment status.

**Conclusion:**

To attain herd immunity against COVID-19, a large proportion of the population needs to be vaccinated and to achieve this the vaccination campaigns should target on specific expectations and motivational factors pertaining to each target group to successfully overcome the challenge of vaccine hesitancy.

**Supplementary Information:**

The online version contains supplementary material available at 10.1186/s12889-021-11620-z.

## Background

The world is going through the biggest pandemic and the most devastating global health crisis of our time and the greatest challenge we have faced since World War II [1]. The current COVID-19 pandemic has affected millions of people around the world, causing more than 3.7 million deaths [2] and large-scale social impact that has impacted everything from healthcare, people’s livelihoods, mental health to enormous economic losses [3–5].

For more than a year the world leaders and public health experts have fought hard and strong to bring an end to the current pandemic by developing an effective vaccine against COVID-19. Worldwide lot of pharmaceutical companies entered the vaccine race to come out with an effective vaccination for COVID-19, this inevitable attention to the vaccines, has made them the unexpected superheroes of 2020 [6].

Clinical trials were conducted and many vaccines like Sinopharm, [7] Pfizer-BioNTech [8], Moderna, [9] Johnson & Johnson [10] etc. have been proven to be effective against COVID-19 with varying levels of effectiveness. According to the WHO at least seven different vaccines across three platforms have been rolled out in countries after the first mass vaccination programme, which started in early December 2020. As of 23rd June 2021, more than 2 billion vaccine doses have been administered across the globe [11]. All over the world vaccinations are happening with full rigor and the UAE is in the top rank of countries with the highest rate of vaccination per capita with more than 5 million people vaccinated [12].

Despite all this, countries around the world are facing challenges to get people vaccinated. There are a lot of speculations and myths about the virus and development of the vaccine against COVID-19 that are being discussed and shared in various media platforms [13]. These factors have caused delays due to unwillingness in people to get vaccinated, leading to vaccine hesitancy. Vaccine hesitancy is referred to “the delay in acceptance or refusal of vaccines despite availability of vaccine services”. The WHO has stated that vaccine hesitancy is a global threat and lack of confidence in vaccination and inconvenience in accessing are the two key factors contributing to hesitancy and unwillingness to get vaccinated [14]. Further based on the R_o_ value for COVID-19, it has been estimated that to achieve herd immunity a vaccination coverage of 75–90% is needed [15]. To achieve this high coverage, it becomes essential to understand the perception of people and their expectation regarding vaccination. Only this will help us foresee and address the vaccination challenges, which is crucial for the success of COVID-19 vaccination to mitigate the impact of the pandemic.

### Objectives

To understand the perception and expectation of COVID-19 vaccination among the UAE population.

To evaluate the factors influencing the acceptance of vaccine against COVID-19 vaccine among the UAE population.

## Materials and methods

The study was approved by the Institutional Review Board (IRB), Department of Health (DOH) Abu Dhabi and all methods were performed in accordance with the relevant guidelines and regulations of the DOH. A cross-sectional study was conducted among the UAE population through an online quantitative survey based on YouGov’s OMNIBUS panel [16] and no personal identifiers were collected by this study. The survey participants were chosen by simple random sampling from a database of 1.2 million across the UAE. The random sampling was done by YouGov’s OMNIBUS panel with pre-set selection criteria which included participants must be residents of the UAE (nationals and expatriates on permanent residency), and the sample should be representative of UAE’s demographics, which included participants from all nationalities, gender, aged 18 years or older. Once the pre-set quota was met no other responder would qualify and will be screened out. The participants were approached through emails or contact numbers. The survey had a screening question to carefully select respondents who on some level felt vaccination offers protection against diseases. The screening question asked participants to evaluate how important they feel about them and their family members receiving vaccination for protection against any disease on a five point Likert scale ranging from “extremely important” to “not important at all”. Participants who felt that vaccination was important were asked further questions in the survey to understand the factors that influence them to accept vaccination. In the UAE, the phase III clinical trial for Sinopharm vaccine against COVID-19 was conducted and at the time of this survey Sinopharm was the only vaccine available in the UAE, therefore questions of this survey were focussed mainly on the understanding and acceptance of Sinopharm vaccine. The survey included questions on (i) demographic variables like age, sex, marital status, place of residence (emirate), income (AED/month) and employment status for audience segmentation, (ii) expectations from COVID-19 vaccine, (iii) motivating factors to get COVID-19 vaccine, (iv) when they would consider getting a COVID-19 vaccination, (v) most trusted channel for getting information on COVID-19 vaccination, (vi) who will they consult before taking a final decision on vaccination, (vii) convincing factors to receive Sinopharm vaccine and (vii) questions pertaining to their awareness and confidence on COVID-19 vaccines and vaccine trials. All questions were closed-ended with multiple response options, the questions on awareness were provided with yes or no options and the questions on confidence about vaccines and clinical trials were assessed on a five-point Likert scale. Respondents completed our survey, and the results of the survey were analysed with various demographical variables of interest.

### Statistical analysis

Descriptive statistics were performed to describe the demographical characteristics of the participants and the perception, expectations, acceptance of COVID−19 vaccine and awareness on vaccines and vaccine trials. They are expressed in percentages (%).

The perception, expectations, acceptance of COVID−19 vaccine and awareness on vaccines and vaccine trials were compared between the groups of demographic variables (gender, age groups, marital status, employment status and income groups), with the chi-square test to analyse the significance of association between the variables and odds ratio (OR) was calculated with 95% confidence interval (95% CI). All data were analysed using the IBM SPSS Statistics Version 28.0.0.0 software.

## Results

The survey was completed by 1003 participants, who belonged to both gender and various nationalities, ages, and income groups, representative of the UAE population demographics. Table 1 shows the distribution of the participants based on the various demographic factors.

**Table 1:** The demographic distribution of the survey participants. (*n* = 1003)

Demographic variables	Groups	Number of participants	Percentage (%)
**Age (in years)**			
	18–24	93	9.3
	25–34	416	41.5
	35–44	325	32.4
	≥ 45	169	16.8
**Gender**			
	Male	651	64.9
	Female	352	35.1
**Nationality**			
	Emirati	77	7.7
	Arab expats	252	25.1
	Asian	572	57
	Europeans/Americans	45	4.5
	Other Nationalities	57	5.7
**Emirates**			
	Abu Dhabi	370	36.9
	Dubai	349	34.8
	Sharjah	158	15.8
	Other Emirates	126	12.6
**Marital status**			
	Married	631	62.9
	Unmarried	372	37.1
**Income (AED/month)**			
	Less than 10,000	484	48.3
	10,000- 25,000	239	23.8
	More than 25,000	182	18.1
	Not specified	98	9.8
**Employment status**			
	Employed	835	83.3
	Unemployed	168	16.7

### Motivation to get COVID-19 vaccination (Fig. 1)

Safety and efficacy of the vaccine emerged as the top driver (52%) for vaccination, followed by not having any major side effects from vaccination (50%), responsibility towards keeping their family safe (47%) and protection against new variants of the virus (47%).

**Fig. 1:**
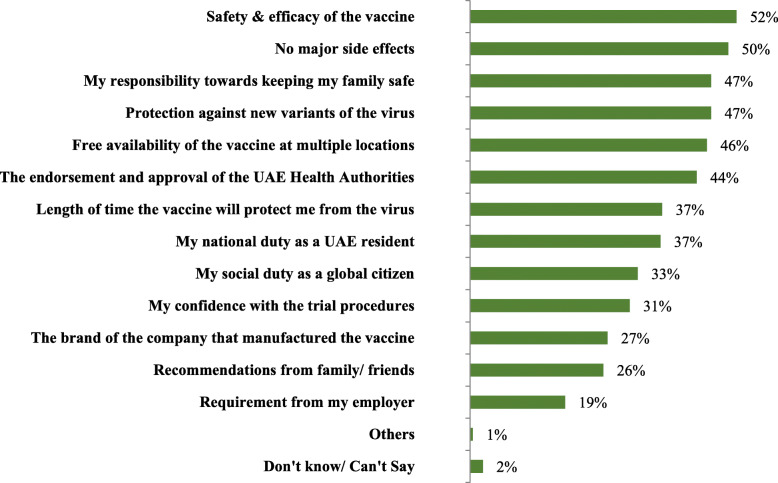
The motivators to get COVID-19 vaccination (*n* = 1003)

### Expectations about COVID-19 vaccination (Fig. 2)

Major expectations of the respondents about the COVID-19 vaccinations were, it should not have any side effects on the body (58%), it should protect themselves and their family (55%) and 52% of the participants also expect it to be free and easily available at multiple locations.

**Fig. 2:**
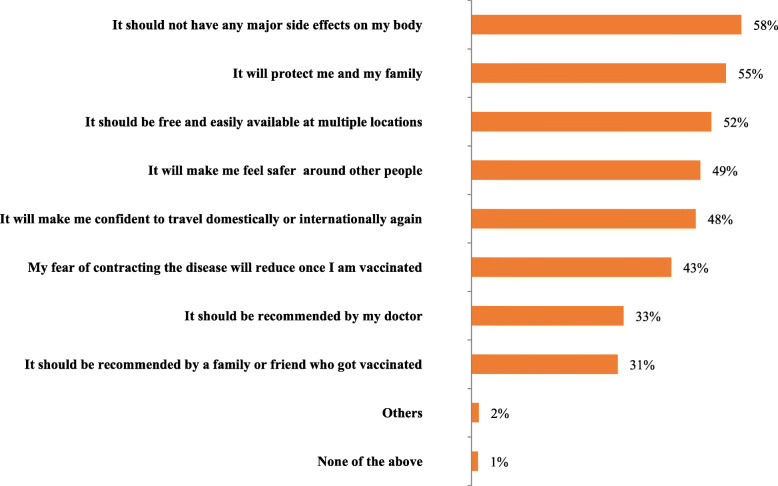
The expectations from COVID-19 vaccination (*n* = 1003)

### Timing of the vaccination (when are people likely to get vaccinated) (Fig. 3)

A quarter of respondents said they would consider getting the vaccine once a reliable source confirms that the vaccine is safe and has no major side effects (26%), while 17% were willing to take it immediately once vaccine is approved and available.

**Fig. 3:**
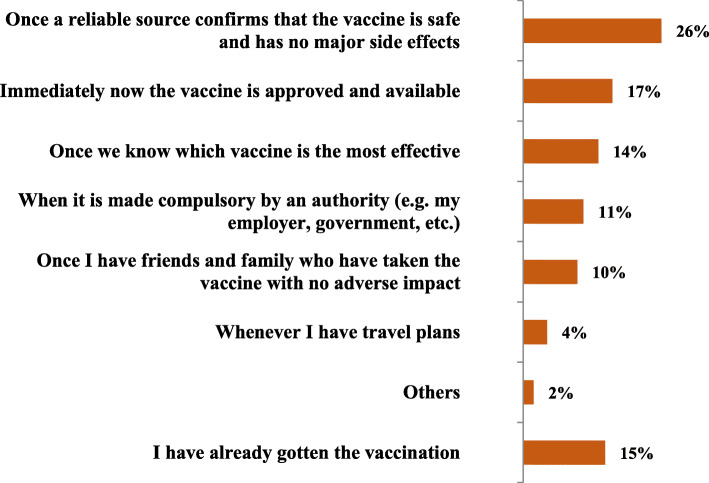
Likelihood of getting a COVID-19 vaccination (*n* = 1003)

### Trustworthy channel for getting information on COVID-19 vaccine (Fig. 4)

Government websites are considered the most trusted channel for getting information on COVID-19 vaccines according to 46% of those surveyed. This is followed by International newspapers/magazines/news channels (22%) and SMS or email from a trusted government source (21%).

**Fig. 4:**
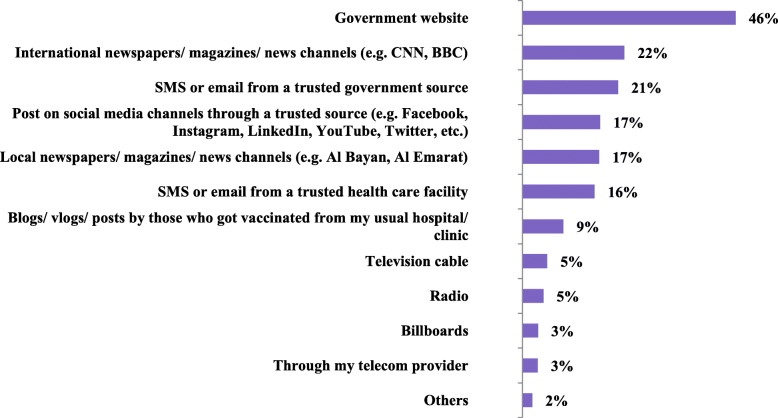
The trustworthy channel for getting information on COVID-19 vaccination (*n* = 1003)

### Consultation before making a final decision to get the COVID-19 vaccine (Fig. 5)

3 out of 5 respondents said they would consult Government health authority advisory websites before making a final decision to get the COVID-19 vaccine. Around two-fifths said they would consult with their family (41%) and their family doctor (37%) first before making a final decision on getting COVID-19 vaccination.

**Fig. 5:**
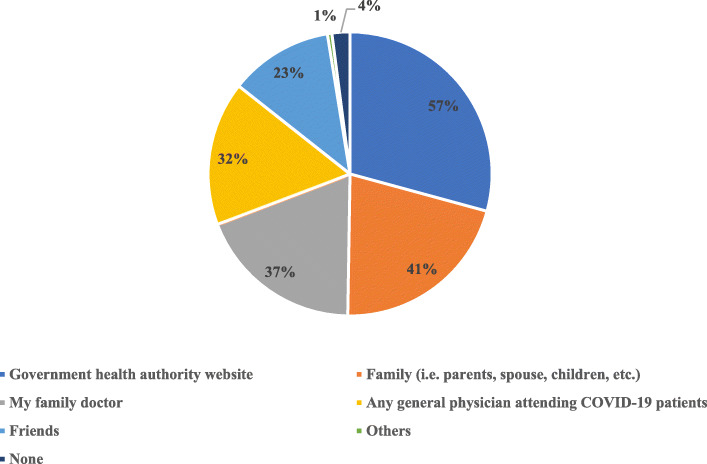
The trusted source for consultation chosen by participants before making a final decision to get the COVID-19 vaccine. (*n* = 1003)

### Trial and vaccine awareness

70% of the participants were aware about the Sinopharm’s inactivated vaccine and 84% were aware about the Phase III clinical trials conducted in the UAE. Over 80% of those surveyed were aware of the Chinese origin of Sinopharm vaccine and 67% were aware that the inactivated vaccine has 79% efficacy against COVID-19. 62% respondents show a high level of confidence with the Phase III trials of the vaccines and 55% show confidence in the Sinopharm vaccine.

### Motivation to get Sinopharm vaccine

Regarding the motivation to take Sinopharm vaccine, its effectiveness against the new strains of COVID-19 emerges as the most convincing factor that would encourage respondents to take the Sinopharm vaccine, as stated by 50% of the participants. This was followed by free vaccinations for all residents (41%) and a decrease in overall cases after the vaccinations roll out (40%).

#### Association of various demographic variables with COVID-19 vaccine perception, expectation, and awareness (supplementary table)

##### Association of gender with vaccine perception, expectations and awareness (supplementary Table 1)

Regarding expectations about the COVID-19 vaccine, more women than men expected the vaccine to reduce the fear of contracting the disease, wanted the vaccine to be recommended by their doctor, wanted the vaccine to be free and easily available at multiple locations and have no major side effects. The differences were found to be statistically significant.

The motivations for getting vaccinated like the responsibility of keeping the family safe, the vaccine being safe and efficient, longer duration of protection offered by the vaccine, no major side effects and protection given by the vaccine against new strains were seen more in females than in males and the difference was again statistically significant. However motivational factors like national duty as a UAE resident were seen more in men than in women, but the difference was not statistically significant.

There was also significant difference in the trusted channel for information regarding vaccination between the two gender. More women than men preferred international newspapers/ magazines/ news channels (e.g., CNN, BBC) and females were twice more likely (p – 0.002, OR – 2.02. 95% CI (1.3–3.1)) to trust blogs/ vlogs/ posts by those who got vaccinated from their usual hospital/ clinic than males. Whereas more males chose information through the telecom provider than females as a trusted channel for information on vaccination. Also, significantly more women than men preferred consultation with family members and any general physician before vaccination.

While documents from health officials explaining the benefits of the vaccination and the evidence of reduction in number of cases after rolling out of vaccination were required for women to take Sinopharm vaccine, men were found to be more confident about the clinical trials and Sinopharm vaccine.

##### Association of age with vaccine perception, expectations, and awareness (supplementary Table 2)

For the analysis of age with vaccine perception, we grouped participants into two age groups less than 35 years and ≥ 35 years. More people in the age group ≥35 years had expectations that the vaccine should protect them and their family, to make them feel safer and confident to travel and lessen the fear of contracting the disease and should be recommended by a doctor than people in age group < 35 years.

Motivational factors for vaccination such as social duty as a global citizen, national duty as a UAE resident, responsibility towards keeping family safe, the brand of the vaccine manufacturer, the safety and efficacy of the vaccine, the vaccine having no major side effects, increased duration of protection and availability at multiple locations were chosen more by people in the higher age group (≥ 35 years), than people in age group < 35 years.

People ≥35 years were more aware about the Sinopharm vaccine and its origin from China than the people in the age group < 35 years, the difference was statistically significant.

People in younger age group(< 35 years) were twice likely to considered billboards as a trusted channel for getting information on vaccination than people in the age group ≥35 years. (P - 0.027).

Almost 20% of the participants in the older group (≥ 35 years) had already got vaccinated compared to 13% in the younger age group (< 35 years) (*p* < 0.01, OR – 1.54, 95% CI (1.1–2.2)).

##### Association of marital status with vaccine perception, expectations, and awareness (supplementary Table 3)

More married people expected the vaccine to be recommended by their doctor than unmarried people. (p - 0.052). National duty as a UAE resident, the safety and efficacy of the vaccine and free availability at multiple locations served as better motivational factors for married people than unmarried people and the differences were statistically significant.

Married people had better awareness about the Sinopharm vaccine and its origin from China and more confident with clinical trials than unmarried people. The difference was statistically significant.

Regarding the trusted channels for information on vaccination, while married men trusted local newspapers/ magazines/ news channels (e.g. Al Bayan, Al Emarat) and SMS or email from a trusted government source, unmarried people chose international newspapers/ magazines/ news channels (e.g. CNN, BBC) and post on social media channels (e.g. Facebook, Instagram, LinkedIn, YouTube, Twitter, etc). This difference was also found to be statistically significant.

##### Association of employment status with vaccine perception, expectations, and awareness (supplementary Table 4)

Our study did not find any significant difference in vaccine perception between working and non-working participants except for working people had more awareness on Sinopharm vaccine, its origin from China and more confident with clinical trials and Sinopharm vaccine. Unemployed people had more concerns on vaccination having no major side effects than employed people (p - 0.009, OR- 1.57, 95% CI (1.13–2.2)).

##### Association of levels of income with vaccine perception, expectations, and awareness (supplementary Table 5)

People with income more than 10,000 AED/ month had more expectations than people with income less than 10,000 AED/month that the vaccine should make them feel safer and confident to travel domestically and internationally, lessen the fear of contracting the disease, and be free and easily available at multiple locations.

Among people with higher income, factors that motivate them to get vaccinated are national duty as a UAE resident, confidence with the clinical trial procedures, the endorsements and the approval of the UAE authorities, the vaccine having no major side effects and protection against new variants of the virus. Also, they had better awareness on Sinopharm vaccine compared to lower income group people. They required independent data proving the effectiveness of the vaccine, number of cases reducing after rolling out the vaccination, research that shows the vaccine is effective on the newer strains of COVID-19, and providing vaccination at home or in clinics without the need to stand in long queues to convince them to take Sinopharm vaccine.

## Discussion

In this study safety of the vaccine emerged as the top motivating factor for vaccination, which is similar to a study done in the United States that showed lesser incidence of major adverse events of the vaccine is associated with higher probability of choosing to get vaccinated from that vaccine [17]. A study in UK on parents’ and guardians’ acceptance of vaccination showed that the main motivation for most people to accept a vaccine for themselves and their children was self-protection against COVID-19 [18].

In our survey, 52% of the people expected the vaccine to be free and easily available at multiple locations. Similar results were shown by another survey done in the UAE which stated 25% of the participants of the survey wanted the vaccine free of cost and 36% were not willing to travel to other Emirates to get vaccination [19].

More than half the respondents indicated that they would consult UAE Health Authority websites before opting for the vaccination, reiterating the fact that Government websites are considered the most trusted channel for vaccine and COVID-19 information. A similar survey in the United States on likelihood of COVID−19 vaccine acceptance showed that an endorsements from the United States Center for Disease Control and Prevention and the World Health Organization were associated with higher probabilities of choosing to take the vaccine [17]. A global survey also showed that respondents who trusted their government were more likely to accept a vaccine than those who said that they did not, with an Odds ratio of 1.67 [20].

In this survey, family and family doctor emerged as the second most trusted source to consult before vaccination. A survey in China also showed that people who valued the doctor’s recommendation as an important factor in deciding to get vaccinated tend to accept the COVID-19 vaccination as soon as possible [21]. This fact was also emphasised in studies, which showed that the attitude of health professional towards vaccines are an important determinant of vaccine uptake for themselves and their likelihood of recommending the vaccine to their patients [22].

Our study showed that females more than males had more worries about safety issues of vaccinations and needed more evidence on the vaccine safety and convincing evidence from reliable sources before getting vaccinated. Similar vaccine survey in France and UK showed that female gender was largely associated with COVID−19 vaccine uncertainty and refusal [23, 24]. This might be because of the fact that many vaccine trials have excluded pregnant and lactating women and therefore the vaccine lacks safety data on this subset of women. This fact may not be reassuring for women who are in their reproductive age group who have concerns over their own as well as their baby’s health [25].

In this study, compared to younger age group (less than 35 years), people who are 35 years and older were more willing to take the vaccine immediately once it was approved and available. Studies from Saudi Arabia and United States showed similar reports that older people were more willing to accept the COVID-19 vaccination than young people [26, 27] However, another survey in the United States, on the other hand showed that younger respondents were more likely to take COVID-19 vaccine and age was found as significant predictor of willingness to take a COVID-19 vaccine [28]. These differences might be because of the different age groups cut off values used in each study.

Married people had more awareness about vaccine trials and relied more on information from trusted government sources than unmarried people and they were more ready to immediately accept vaccination once approved. A similar survey in China showed that being married increase the probability of accepting COVID-19 vaccination [21]. A study in Saudi Arabia on determinants of COVID-19 vaccine acceptance showed that married people were significantly associated with vaccine acceptance [26].

Our study shows that people who are above 35 years old and who are married had more awareness or were more willing to take the vaccination, this could be because of the fact that most people who were 35 years and above were also married and this age group is more responsible and proactive when it comes to their health or the health of their family. Furthermore, most public messaging has focused on the severity and threat of COVID-19 to older adults and this had made young adults less worried about the seriousness of the disease [29].

Awareness about vaccination was higher in working people compared to non-working people and concerns on side effects were seen more in unemployed people. A survey on vaccine hesitancy in France also showed that vaccine hesitancy was lower in working individuals than with non-working individuals [23]. This again might be because of the fact that working people are travelling and in close contact with other co-workers therefore more at risk of COVID−19 which could be a motivation to know more about vaccination and other preventive measures.

The survey showed increased awareness about clinical trials and about the Sinopharm vaccine among people in the higher income group and this group was more confident about the clinical trials. These are important factors for predicting vaccine acceptance. Similar reports were seen in the global survey on vaccine acceptance [20] and in a survey in the United States that showed vaccine acceptance increased with increasing income level [30]. The UK survey also showed that low-income groups were one of the largest predictors of COVID-19 vaccine uncertainty and refusal [24]. This could be due to the fact that people in the higher socio-economic status also have better educational status and hence better awareness about vaccination trials and vaccines. However, further understanding of these facts in a sociocultural context is necessary, as studies were not able to find consistent association between education and vaccine hesitancy [31].

Conspiracy theories on vaccination, especially on the safety of vaccine, vaccine affecting fertility and pregnancy, the infamous microchip theory and several others could have also contributed to the variation in vaccine acceptance among different age group, gender and income group [32]. Further qualitative research is required to understand the role of such theories in contributing towards vaccine hesitancy.

### Insights based on the survey findings to improve vaccine acceptance and vaccination rate

This survey gives an insight on information gaps, perceptions, and the basis of vaccine hesitancy. This is useful for designing targeted strategies with respect to the age, gender, marital status, household income, and employment status of populations to augment public health efforts.

From our survey findings people residing in the UAE have increased likelihood to receive vaccinations when vaccines are endorsed by trusted government health authorities, recommended by physicians and family doctors, and merits effectively communicated through government websites and trusted news channels. Likelihood of vaccination is further increased by availability of vaccines at multiple sites and by providing vaccination free of charge. Emphasizing vaccine safety and efficacy, as well as reiterating the fact about the vaccine having no major side effects is effective at reducing vaccine hesitancy, particularly in women, a key demographic of vaccine hesitancy around the globe. Findings from our study show that social media posts (e.g., from Facebook, Twitter, LinkedIn, etc.) are an effective mode of communication to reach unmarried people and billboard communications are likely to be effective in reaching people, particularly in the younger age group (< 35 years). Effective strategies that ensure the safety and efficiency of vaccination and instil confidence in clinical trials and vaccines targeting unemployed and lower income (< 10,000AED/month) populations are needed to improve acceptance of vaccination against COVID-19.

### Strengths and limitations

The study helps us understand the perception of people about COVID-19 vaccination, which gives an insight on what residents in the UAE expect, what they need to get vaccinated and what are the trusted channels through which we can communicate and target our vaccine campaigns. This understanding will help to device strategies to improve vaccination.

However, there are a few limitations in this study. The survey has not taken into consideration the perception of participants who thought vaccination was not important, as the study aimed to understand the factors which had motivated and influenced people to consider vaccines important. We focused on this group to identify communication strategies to increase uptake of COVID-19 vaccination. This limitation may have affected the representativeness of the sample and complete understanding of certain factors. Also, the results are self-reported, which may reflect individual and response bias and may not reflect the true practice. In future studies, it would be enlightening to also understand which factors contributed to vaccine mistrust.

## Conclusion

We conclude that the perception and expectations regarding vaccination against COVID-19 varies with age, gender, marital status, income, and employment status. Public health strategies to improve vaccine acceptance and vaccine coverage should address these concerns pertaining to women, young and unmarried individuals, people who are unemployed and who belong to lower income levels to attain effective herd immunity in the population.

## Supplementary Information

**Additional file 1: Supplementary Table 1.** Association of Gender with vaccine survey outcomes.

**Additional file 2: Supplementary Table 2.** Association of age group with vaccine survey outcomes.

**Additional file 3: Supplementary Table 3.** Association of Marital status with vaccine survey outcomes.

**Additional file 4: Supplementary Table 4.** Association of employment status with vaccine survey outcomes.

**Additional file 5: Supplementary Table 5.** Association of income levels with vaccine survey outcomes.

## Data Availability

The data is available with the first author and will be produced on request.

## Abbreviations

SARS-CoV-2: Severe acute respiratory syndrome coronavirus 2
OR: Odds ratio
CI: Confidence interval
