# Supplementary material for: Understanding perception and acceptance of Sinopharm vaccine and vaccination against COVID–19 in the UAE
Source: BMC Public Health. 2021 Aug 30;21:1602. doi: 10.1186/s12889-021-11620-z (PMC8404750; doi:10.1186/s12889-021-11620-z)
Supplement: Supplementary file 1 — Additional file 1: Supplementary Table 1. Association of Gender with vaccine survey outcomes. [file 12889_2021_11620_MOESM1_ESM.docx]

**Supplementary Table 1: Association of Gender with vaccine survey outcomes**

| **Question** | **Male** | **Female** | **Odds ratio**  **(95% CI)** | **P value** |
| --- | --- | --- | --- | --- |
| **Expectations from COVID-19 vaccination** | | | | |
| My fear of contracting the disease will reduce once I am vaccinated. | 39.8 | 49.4 | 1.4  (1.1 – 1.9) | 0.003 |
| It should be recommended by my doctor. | 29.3 | 40.1 | 1.6  (1.2 – 2.1) | <0.001 |
| It should be free and easily available at multiple locations | 48.1 | 56.3 | 1.3  (1.1 – 1.8) | 0.014 |
| I should not have major side effects in my body | 50.5 | 63.4 | 1.7  (1.3 – 2.2) | <0.001 |
| **Motivation factors for getting the COVID-19 vaccination** | | | | |
| My responsibility Keeping my family safe | 43.6 | 52.3 | 1.4  (1.1 – 1.3) | <0.010 |
| Safety and Efficacy of the vaccine | 46.2 | 59.4 | 1.7  (1.3 - 2.2) | <0.010 |
| Length of the time the vaccine will protect me from virus. | 33.8 | 41.5 | 1.4  (1.1 – 1.8) | 0.016 |
| No major side effects | 44.4 | 57.4 | 1.7  (1.3 – 2.1) | <0.010 |
| Protection against new variants of the virus | 41.3 | 54.5 | 1.7  (1.3 – 2.2) | <0.010 |
| Free availability of the vaccine at multiple locations | 41.6 | 53.1 | 1.6  (1.2 – 2.1) | <0.010 |
| **Trusted channel for getting information on COVID-19 vaccination** | | | | |
| International newspapers/ magazines/ news channels (e.g., CNN, BBC) | 20.7 | 26.4 | 1.4  (1.01 – 1.8) | 0.048 |
| Through my telecom provider | 4.1 | 1.1 | 3.71  (1.27 – 10.5) | 0.007 |
| Blogs/ vlogs/ posts by those who got vaccinated from my usual hospital/ clinic | 6.8 | 12.8 | 2.02  (1.3- 3.1) | 0.002 |
| **Consultation before taking a final decision on vaccination against COVID-19** | | | | |
| Family (i.e., parents, spouse, children, etc.) | 36.7 | 49.1 | 1.6  (1.3 – 2.1) | <0.001 |
| Any general physician attending COVID-19 patients | 29.6 | 38.6 | 1.5  (1.1- 1.9) | 0.005 |
| **Confidence on clinical trials on COVID-19 vaccination** | | | | |
| I am very confident with the clinical Phase III trials of the COVID19 vaccines? | 31.79 | 17.89 | 1.78  (1.3-2.4) | <0.001 |
| **Confidence on Sinopharm vaccine** | | | | |
| I am very confident with the Sinopharm vaccine? | 26.26 | 15.05 | 1.74  (1.2-2.1) | <0.001 |
| **Factors that would convince you to take the Sinopharm vaccine** | | | | |
| Documentary from health officials explaining the benefits of the vaccine | 31.2 | 42.3 | 1.6  (1.2 – 2.1) | <0.010 |
| Total number of cases going down gradually after the vaccination roll out | 33.9 | 49.1 | 1.9  (1.4 – 2.4) | <0.010 |
