# Supplementary material for: Understanding perception and acceptance of Sinopharm vaccine and vaccination against COVID–19 in the UAE
Source: BMC Public Health. 2021 Aug 30;21:1602. doi: 10.1186/s12889-021-11620-z (PMC8404750; doi:10.1186/s12889-021-11620-z)
Supplement: Supplementary file 2 — Additional file 2: Supplementary Table 2. Association of age group with vaccine survey outcomes. [file 12889_2021_11620_MOESM2_ESM.docx]

**Supplementary Table 2 : Association of age group with vaccine survey outcomes**

| **Question** | **18-34 years** | **≥ 35 years** | **Odds ratio**  **(95% CI)** | **P value** |
| --- | --- | --- | --- | --- |
| **Expectations from COVID-19 vaccination** | | | | |
| It will protect me and my family | 49.1 | 59.9 | 1.5  (1.2 - 1.9) | P<0.001 |
| It will make me feel safer around other people | 45.8 | 52.2 | 1.2  (1.01 – 1.6) | 0.043 |
| It will make me confident to travel domestically or internationally again | 43.8 | 53.0 | 1.4  (1.1 – 1.8) | 0.004 |
| My fear of contracting the disease will reduce once I am vaccinated | 38.7 | 47.8 | 1.4  (1.1 – 1.8) | 0.004 |
| It should be recommended by my doctor | 29.1 | 37.2 | 1.4  (1.1 – 1.8) | 0.007 |
| It should not have any major side effects on my body | 50.9 | 59.3 | 1.4  (1.1 – 1.8) | 0.008 |
| **Motivation factors for getting the COVID-19 vaccination** | | | | |
| My social duty as a global citizen | 25.7 | 38.3 | 1.7  (1.3-2.3) | <0.001 |
| My national duty as a UAE resident | 30.5 | 43.7 | 1.7  (1.3 – 2.3) | <0.001 |
| My responsibility towards keeping my family safe | 41.3 | 52.2 | 1.5  (1.2 – 1.9) | <0.001 |
| The brand of the company that manufactured the vaccine | 23.8 | 30.2 | 1.3  (1.04 – 1.8) | 0.023 |
| My confidence with the trial procedures | 25.9 | 35.6 | 1.5  (1.2 – 2.1) | <0.001 |
| Safety & efficacy of the vaccine | 44.6 | 57.3 | 1.6  (1.2 – 2.1) | <0.001 |
| Length of time the vaccine will protect me from the virus | 31.8 | 41.3 | 1.5  (1.1 -1.9) | <0.001 |
| No major side effects | 45.8 | 52.2 | 1.2  (1.01 – 1.7) | 0.043 |
| Free availability of the vaccine at multiple locations | 42.4 | 49.0 | 1.3  (1.01 – 1.7) | 0.042 |
| **Trusted channels for getting information on COVID-19 vaccination** | | | | |
| Billboards | 4.9 | 2.2 | 2.2  (1.1-4.6) | 0.027 |
| **Consultation before taking a final decision on vaccination against COVID-19** | | | | |
| My family doctor | 33 | 41.1 | 1.4  (1.1 – 1.8) | 0.009 |
| **Awareness about Sinopharm vaccine** | | | | |
| Are you aware of Sinopharm's inactivated vaccine? | 67.8 | 74.3 | 1.3  (1.04 – 1.8) | 0.026 |
| The Sinopharm inactivated vaccine has its origin from China | 80.2 | 86.6 | 1.6  (1.14 – 2.3) | 0.007 |
| The Sinopharm's inactivated vaccine has 79% efficacy against COVID-19 | 64.2 | 73.1 | 1.5  (1.2 – 1.9) | 0.003 |
| **Factors that would convince you to take the Sinopharm vaccine** | | | | |
| Research that shows vaccine is effective on the new strains of COVID19 | 43.2 | 52 | 1.4  (1.1 – 1.8) | 0.005 |
