# Supplementary material for: Understanding perception and acceptance of Sinopharm vaccine and vaccination against COVID–19 in the UAE
Source: BMC Public Health. 2021 Aug 30;21:1602. doi: 10.1186/s12889-021-11620-z (PMC8404750; doi:10.1186/s12889-021-11620-z)
Supplement: Supplementary file 3 — Additional file 3: Supplementary Table 3. Association of Marital status with vaccine survey outcomes. [file 12889_2021_11620_MOESM3_ESM.docx]

**Supplementary Table 3 : Association of Marital status with vaccine survey outcomes**

| **Questions** | **Married**  **(%)** | **Single**  **(%)** | **Odds ratio**  **(95% CI)** | **P value** |
| --- | --- | --- | --- | --- |
| **Motivation factors for getting the COVID-19 vaccination** | | | | |
| My national duty as a UAE resident | 40.4 | 31.2 | 1.5  (1.1 – 1.9) | 0.004 |
| Safety & efficacy of the vaccine | 54.2 | 45.2 | 1.4  (1.1 – 1.8) | 0.006 |
| Free availability of the vaccine at multiple locations | 48.3 | 33.4 | 1.3  (1.03 – 1.7) | 0.030 |
| **Trusted channels for getting information on COVID-19 vaccination** | | | | |
| Local newspapers/ magazines/ news channels (e.g., Al Bayan, Al Emarat) | 20 | 14.2 | 1.5  (1.1 – 2.1) | 0.026 |
| International newspapers/ magazines/ news channels (e.g., CNN, BBC) | 20.6 | 26.3 | 1.3  (1.03 – 1.8) | 0.042 |
| SMS or email from a trusted government source | 22 | 16.1 | 1.4  (1.1- 2.1) | 0.027 |
| Post on social media channels through a trusted source (e.g., Facebook, Instagram, LinkedIn, YouTube, Twitter, etc.) | 14.7 | 20.7 | 1.5  (1.1 – 2.1) | 0.018 |
| **Consultation before taking a final decision on vaccination against COVID-19** | | | | |
| My family doctor | 42.8 | 27.2 | 2.0  (1.5 – 2.6) | <0.001 |
| **Awareness about Sinopharm vaccine** | | | | |
| Are you aware of Sinopharm's inactivated vaccine? | 77.3 | 60.2 | 2.2  (1.7 – 2.9) | <0.001 |
| The Sinopharm inactivated vaccine has its origin from China | 85.4 | 79.8 | 1.5  (1.05–2.07) | 0.028 |
| **Confidence on clinical trials on COVID-19 vaccination** | | | | |
| How confident would you say you are with the clinical Phase III trials of the COVID19 vaccines? | 30.6 | 20.7 | 1.48  (1.1-1.9) | <0.001 |
