# Supplementary material for: Understanding perception and acceptance of Sinopharm vaccine and vaccination against COVID–19 in the UAE
Source: BMC Public Health. 2021 Aug 30;21:1602. doi: 10.1186/s12889-021-11620-z (PMC8404750; doi:10.1186/s12889-021-11620-z)
Supplement: Supplementary file 4 — Additional file 4: Supplementary Table 4. Association of employment status with vaccine survey outcomes. [file 12889_2021_11620_MOESM4_ESM.docx]

**Supplementary Table 4 : Association of employment status with vaccine survey outcomes**

| **Question** | **Working** | **Not working** | **Odds ratio**  **(95% CI)** | **P value** |
| --- | --- | --- | --- | --- |
| **Motivation factors for getting the COVID-19 vaccination** | | | | |
| No major side effects | 47.1 | 58.3 | 0.63  (0.45 – 0.88 | 0.009 |
| The Sinopharm inactivated vaccine has its origin from China | 84.6 | 76.8 | 1.6  (1.1 – 2.5) | 0.017 |
| The Sinopharm's inactivated vaccine has 79% efficacy against COVID-19 | 70.1 | 61.3 | 1.4  (1.04 – 2.08) | 0.029 |
| Confidence on clinical trials on COVID-19 vaccination | | | | |
| How confident would you say you are with the clinical Phase III trials of the COVID19 vaccines? | 27.90 | 22.20 | 1.7  (1.2-2.2) | <0.001 |
| Confidence on Sinopharm vaccine | | | | |
| How confident would you say you are with the Sinopharm vaccine? | 23.71 | 15.4 | 1.6  (1.2-2.2) | <0.001 |
| **Factors that would convince you to take the Sinopharm vaccine** | | | | |
| Documentary from health officials explaining the benefits of the vaccine | 36.8 | 26.8 | 1.5  (1.1 – 2.3) | 0.008 |
