# Supplementary material for: Understanding perception and acceptance of Sinopharm vaccine and vaccination against COVID–19 in the UAE
Source: BMC Public Health. 2021 Aug 30;21:1602. doi: 10.1186/s12889-021-11620-z (PMC8404750; doi:10.1186/s12889-021-11620-z)
Supplement: Supplementary file 5 — Additional file 5: Supplementary Table 5. Association of income levels with vaccine survey outcomes. [file 12889_2021_11620_MOESM5_ESM.docx]

**Supplementary Table 5 : Association of income levels with vaccine survey outcomes**

| **Question** | **Income ≤ 10,000 AED/month** | **Income > 10,000 AED/month** | **Odds ratio**  **(95% CI)** | **P value** |
| --- | --- | --- | --- | --- |
| Expectations from COVID-19 vaccination | | | | |
| It will make me confident to travel domestically or internationally again | 43.8 | 55.6 | 1.6  (1.2 – 2.1) | <0.001 |
| My fear of contracting the disease will reduce once I am vaccinated | 40.7 | 47.7 | 1.3  (1.02 – 1.7) | 0.037 |
| It should be free and easily available at multiple locations | 45.7 | 57.0 | 1.5  (1.2 – 2.1) | <0.001 |
| It should not have any major side effects on my body | 48.8 | 61.3 | 1.6  (1.2 – 2.1) | <0.001 |
| **Motivation factors for getting the COVID-19 vaccination** | | | | |
| My national duty as a UAE resident | 31.2 | 43.7 | 1.7  (1.3 – 2.2) | <0.001 |
| My confidence with the trial procedures | 24.8 | 37.5 | 1.8  (1.1 – 2.4) | <0.001 |
| The endorsement and approval of the UAE Health Authorities | 39.7 | 48.5 | 1.4  (1.2 – 1.8) | 0.009 |
| No major side effects | 43.2 | 54.4 | 1.5  (1.2 – 2.04) | <0.001 |
| Protection against new variants of the virus | 43.6 | 50.4 | 1.3  (1.01 – 1.7) | 0.045 |
| **Consultation before taking a final decision on vaccination against COVID-19** | | | | |
| My family doctor | 33.7 | 42.0 | 1.4  (1.1 – 1.9) | 0.011 |
| **Awareness about Sinopharm vaccine** | | | | |
| Are you aware of Sinopharm's inactivated vaccine? | 64 | 81.5 | 2.4  (1.8 – 3.4) | <0.001 |
| Were you aware of the clinical trials taking place in the UAE for vaccinations? | 82.9 | 90 | 1.8  (1.2 – 2.7) | 0.002 |
| The Sinopharm inactivated vaccine has its origin from China | 81.4 | 86.9 | 1.5  (1.1 – 2.2) | 0.029 |
| **Factors that would convince you to take the Sinopharm vaccine** | | | | |
| Independent data to prove the effectiveness of the vaccine | 27.9 | 45.1 | 2.1  (1.6 – 2.8) | <0.001 |
| Total number of cases going down gradually after the vaccination roll out | 34.5 | 44.9 | 1.5  (1.2 – 2.0) | 0.002 |
| Vaccinations to be provided at home or clinics without the need to stand in long queues | 22.5 | 33.0 | 1.7  (1.3 – 2.3) | <0.001 |
| Research that shows vaccine is effective on the new strains of COVID19 | 43.4 | 52.0 | 1.4  (1.1 – 1.8) | 0.011 |
